# Functional annotation of non-coding variants identifies a novel enhancer with activity in neural crest cell–derived lineages

**DOI:** 10.21203/rs.3.rs-9741689/v1

**Published:** 2026-06-05

**Authors:** Vartika Bisht, Neil Slaven, Miriam Smits, Ludo Pagie, Tijn van Balen, Jihed Chouaref, Wilhelmina S. Kerstjens-Frederikse, Cleo C. van Diemen, Jeroen Korving, Harry Begthel, Len A. Pennacchio, Joris van Arensbergen, Alexey V. Pindyurin

**Affiliations:** Annogen BV; Lawrence Berkeley National Laboratory; Annogen BV; Annogen BV; Annogen BV; Annogen BV; University Medical Centre Groningen; University Medical Centre Groningen; Hubrecht Institute – KNAW and University Medical Center Utrecht; Hubrecht Institute – KNAW and University Medical Center Utrecht; Lawrence Berkeley National Laboratory; Annogen BV; Annogen BV

**Keywords:** Non-coding variants, cardiac-specific enhancer, neural crest cells, HES1, MPRA, SuRE assay, congenital heart disease

## Abstract

Congenital heart disease (CHD) is one of the most prevalent and severe structural birth defects, affecting approximately 1% of live births. Because a substantial proportion of CHD patients do not harbor pathogenic mutations in protein-coding regions, non-coding variants are thought to underlie many unexplained cases. However, predicting the functional consequences of non-coding variation from sequence alone remains challenging due to our limited understanding of how regulatory information is encoded in the genome. Here, we used the Survey of Regulatory Elements (SuRE) assay to functionally screen 4.7 million non-coding variants from six individuals with an unexplained genetic predisposition to CHD in cultured AC16 human cardiomyocytes, identifying 18,201 regulatory quantitative trait loci (raQTLs). Systematic prioritization highlighted rs74330989, leading to the discovery of a novel cardiac enhancer, hs3112. Using a LacZ transgenic mouse assay, we demonstrate that hs3112 functions as a robust cardiac enhancer at embryonic day (E) 11.5, with predominant activity in neural crest cell (NCC)-derived populations, as demonstrated by co-staining with an anti-Sox10 antibody. In particularly, enhancer activity was detected in the outflow tract (OFT). Notably, introduction of the alternate rs74330989 allele completely abolished enhancer activity. Collectively, these results provide functional validation of the non-coding variant rs74330989 and demonstrate its requirement for hs3112 enhancer activity. As hs3112 is predicted to regulate *HES1*, which encodes a transcription factor essential for cardiogenesis, our findings suggest a direct mechanistic link between non-coding variation and CHD pathogenesis.

## Introduction

Cardiogenesis is a highly coordinated and tightly regulated sequence of events that begins early in embryonic development and progresses through multiple critical stages(1). Elucidating the molecular networks governing this cascade is essential for understanding both normal cardiac morphogenesis and the origins of congenital heart diseases (CHDs). CHDs represent some of the most prevalent and severe birth defects, affecting ~ 1% of live births(2). Their clinical spectrum ranges from relatively mild anomalies, such as septal defects, to complex and life-threatening malformations, including tetralogy of Fallot and hypoplastic left heart syndrome (HLHS)(3).

Among the cellular populations that drive cardiac morphogenesis, cardiac neural crest cells (cNCCs)(4)^,^ (5)^,^(6) are of particular importance. These cells contribute to the development of the pharyngeal arch arteries, cardiac outflow tract (OFT), valves, and the interventricular septum(7). cNCCs originate from a migratory, multipotent subset of neural crest cells (NCCs) that arise in the dorsal-most region of the nascent central nervous system (CNS)(8). More specifically, NCCs emerging between the mid-otic placode and the third somite delaminate from the dorsal neural tube, migrate toward the cardiac primordium, and undergo lineage-restricted differentiation, at which stage they are designated as cNCCs(4)^,^(5)^,^(6). Consistent with their essential developmental roles, disruptions in NCC specification, migration, or differentiation have been strongly implicated in CHD pathogenesis(9)^,^(10). For example, mice carrying a C4232R missense mutation in Low density lipoprotein receptor-related protein 1 (Lrp1) exhibit atrioventricular septal defects and double outlet right ventricle. Targeted expression of this mutant allele specifically in cNCCs fully reproduces the cardiac abnormalities(11), demonstrating a cell-autonomous requirement for Lrp1 in this lineage. Together, these observations highlight the critical contribution of NCC biology to congenital cardiac malformations.

Although advances in genetic screening, particularly whole exome sequencing (WES) and targeted gene panels, have improved the identification of coding mutations, these approaches still fail to pinpoint causative variants in a substantial proportion of CHD cases(12). Consequently, attention has increasingly shifted towards the non-coding genome. Indeed, genome-wide association studies (GWASs) have emphasized the importance of non-coding variation in CHD, showing that more than 90% of CHD-associated SNPs lie within non-coding regions(13). While whole genome sequencing (WGS) provides the most comprehensive strategy to detect non-coding variants with potential regulatory impact, identifying candidate variants is insufficient; functional validation within a relevant developmental context remains the critical bottleneck.

This issue is partly being addressed by recent advances in high-throughput functional genomics, which enable the systematic, large-scale characterization of regulatory elements(14). For example, a recent functional lentiviral massively parallel reporter assay (lentiMPRA) performed in human iPSC-derived cardiomyocytes (CMs) screened 6,590 non-coding de novo variants (ncDNVs) from CHD trios and identified 403 variants that significantly alter cis-regulatory element (CRE) activity(15). Importantly, studies indicate that pathogenic non-coding variants often disrupt transcription factor (TF) binding or otherwise modify transcriptional regulatory activity. For instance, a variant in the *HES1* promoter (rs148941464) was shown to impair RXRA binding, leading to aberrant *HES1* overexpression and an increased risk of transposition of the great arteries(16). Consistently, a meta-analysis of 14,784,017 variants across four CHD cohorts (n = 55,342) identified 16 novel loci associated with the disease, including rs189203952, which is predicted to disrupt the binding sites of four cardiac TFs in the *SPAG9* promoter and is associated with left ventricular OFT obstruction(17). Additionally, variants within an intronic regulatory region of *NOTCH1* have been associated with an increased risk of left ventricular OFT obstructive defects(18). Collectively, these findings demonstrate that recent methodological advances enable the effective identification of non-coding CHD variants that drive pathogenesis by disrupting TF binding and regulatory element activity.

Among these approaches, the Survey of Regulatory Elements (SuRE) assay provides a powerful platform for large-scale quantification of non-coding regulatory activity(19). In this study, we applied SuRE to to the full genomes of individuals with a high genetic predisposition to CHD, whose conditions remained unresolved by WES or targeted gene panel sequencing. By functionally annotating over 4.7 million variants across six such genomes, we identified 18,201 reporter assay quantitative trait loci (raQTLs). After stringent filtering and prioritization, we focused on raQTL rs74330989, which led us to a previously uncharacterized putative enhancer, hs3112. Using complementary in vitro and in vivo assays, we demonstrate that hs3112 functions during early heart development and may regulate *HES1*, a gene critical for cNCC function. Together, our work provides a large resource comprising millions of functionally annotated non-coding variants and identifies a previously unrecognized heart-specific regulatory element hs3112, whose activity in vivo is abolished by a mutation at rs74330989.

## Results

### Testing 4.7 million genomic variants in a CM cell line for their functional impact on cis-regulatory elements

**Figure 1:**
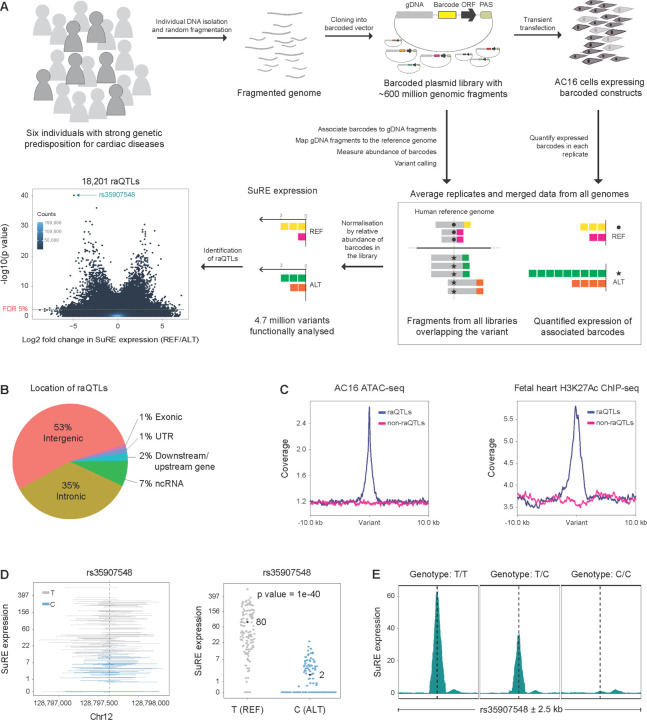
Identification and functional characterisation of cardiac-specific raQTLs. (**A**) Experimental design and variant identification. Schematic overview of the SuRE assay utilized to functionally assess genomic variants (SNPs and InDels) for regulatory activity in the adult heart-derived AC16 human CM cell line. Out of 4.7 million non-coding variants analysed, 18,201 raQTLs were identified. (**B**) Genomic context of raQTLs. Distribution of major genomic features (e.g., introns, intergenic regions,exons) overlapping the identified raQTLs. (**C**) Epigenomic enrichment of raQTLs. raQTLs are significantly enriched in active chromatin markers,ATAC-seq signals in AC16 cells (left) and H3K27Ac ChIP-seq signals in human fetal hearts (right). Epigenomic data were sourced from(37). (**D**) Functional impact of a lead raQTL. DNA fragments overlapping SNP rs35907548 are color-coded byallele and arranged according to their measured SuRE expression levels (left). The corresponding violin plot (right) summarizes these results, showing that fragments carrying the ALT allele display significantly reduced regulatory activity compared to those with the REF allele. (**E**) Dose-dependent effect of rs35907548. SuRE signal profiles from three studied patient genomesdiffering in their base composition at rs35907548 (REF allele T, ALT allele C). The presence of the ALT C allele proportionally decreases the regulatory activity, leading to its almost complete absence in the homozygous alternate individual. (**A-E**) gDNA, genomic DNA fragment; ORF, open reading frame; PAS, polyadenylation signal; FDR, false discovery rate.

To screen for functional regulatory variants that may play a role in the development or function of the human heart, we utilized the previously described high-throughput Survey of Regulatory Elements (SuRE) assay(19). We used genomic DNA samples from a cohort of individuals genetically predisposed to heart diseases as input, reasoning that such genetic material would be enriched for regulatory variants of interest. Briefly, a group of six patients or their close relatives, all from families with several cases of severe heart defects (Supplementary Fig. 1A) was preselected from a larger, unexplained cohort, in which no pathogenic variants had been identified in the coding portion of the genome(20) (see Materials and Methods for details). Next, genomic DNA isolated from each selected individual was sheared into fragments centered around 400–600 bp and subcloned along with a random 20-bp barcode sequence in a promoter-less vector upstream of a *GFP* reporter gene. This process yielded six highly complex plasmid libraries, each containing approximately 600 million clones (Fig. 1A; Supplementary Fig. 1B). Next-generation sequencing (NGS) was performed on libraries to (i) map the genomic locations and identify the variants within these fragments, (ii) associate both reference (REF) and alternate (ALT) allele fragments with unique barcodes, and (iii) measure the abundance of each barcode (Supplementary Table 1). Concurrently, each plasmid library was transiently transfected in at least two replicates into cultured AC16 human CMs(21). The abundance of barcodes in the resulting mRNA was quantified by NGS (Supplementary Table 1). The mRNA barcode counts were then averaged across replicates and normalized to account for the barcode abundances in the plasmid libraries. This comprehensive approach allowed us not only to generate genome-wide SuRE profiles, which highlight regulatory elements (Supplementary Fig. 2), but also to quantitatively assess the regulatory potential of 4.7 million variants (Fig. 1A).

We defined variants as raQTLs if they met the following two criteria: (i) at least three-fold difference in SuRE signal between the REF and ALT alleles, and (ii) a false discovery rate (FDR) cutoff of 0.05 for p values based on a two-sided Wilcoxon rank-sum test. In total, 18,201 raQTLs were identified (Fig. 1A), of which ~ 96% were SNPs and ~ 4% were InDels. Each raQTL was, on average, covered by about 320 genomic fragments (Supplementary Fig. 1C), and the mean SuRE signal of raQTLs was five-fold higher than for non-raQTLs (Supplementary Fig. 1D). Similar to all assessed variants, raQTLs were primarily located in the non-coding portion of the genome, specifically in intergenic regions and introns (Fig. 1B; Supplementary Fig. 1E). Furthermore, compared to non-raQTLs, raQTLs demonstrated a high enrichment in open and active chromatin features in both AC16 cells and human fetal heart tissue (Fig. 1C). The raQTL with the lowest p value, rs35907548, showed a 40-fold reduction in the SuRE signal for the ALT allele compared to the REF allele (Fig. 1A,D). This reduction was also evident in the SuRE enrichment profiles of individuals with heterozygous and homozygous ALT alleles. Notably, the individual homozygous for the ALT allele exhibited an almost complete absence of the SuRE peak (Fig. 1E). Interestingly, the rs35907548 REF allele has previously been associated with increased regulatory activity in data from the cis-eQTLGen consortium, which evaluate the effects of genetic variation on gene expression in blood samples from 31,684 individuals(22). Taken together, our comprehensive analysis identified thousands of predominantly non-coding variants with a significant regulatory impact in a model human AC16 CM cell line. A web-based tool, SuREVizHeart, for exploring and visualizing the functional impact of all studied non-coding genetic variants is available at: http://195.114.233.102:3838/.

### raQTLs are highly enriched for cell type relevant TF binding sites

Previous studies have established that the regulatory regions detected by SuRE are dependent on the cell type in which the library is assayed; some identified elements are cell type-specific, while others are shared across different cell types(19). Extending these observations, SuRE identified regulatory regions in the AC16 CM cell line, which are enriched with H3K27Ac marks in fetal hearts (Supplementary Fig. 2A) compared to non-relevant tissues, such as fetal limbs. Some of the AC16 raQTLs overlap with heart enhancers previously validated in vivo using a CRISPR/Cas9-mediated transgenic mouse assay called enSERT(23) (Supplementary Fig. 2B–D).

**Figure 2:**
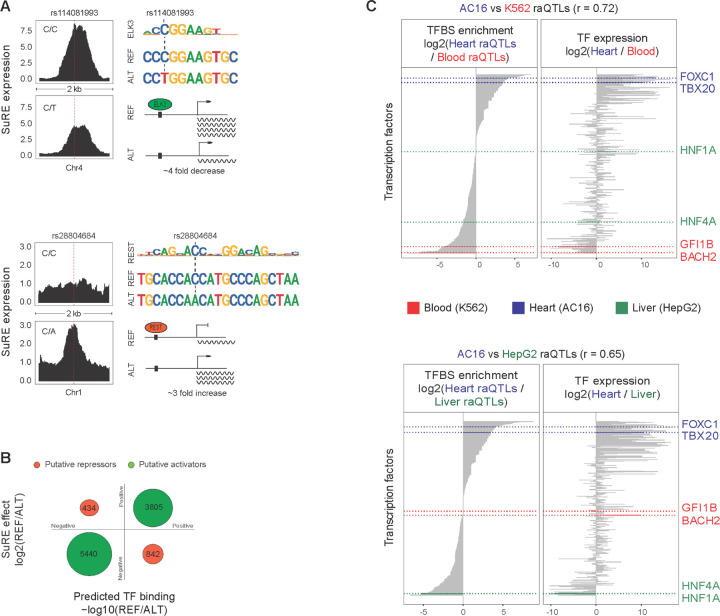
Correlation between raQTLs, predicted TF binding, and regulatory activity. (**A**) Allele-specific regulatory activity and predicted TF binding changes. Comparison of SuRE signals from individuals homozygous (REF/REF) and heterozygous (REF/ALT) for selected raQTLs versus the predicted changes in TF binding affinity (predicted using JASPAR CORE 2024(25)). Examples shown include ELK3 and REST, both known to function as activators and repressors depending on the cellular context. (**B**) Concordance between functional assays and binding predictions. A contingency table systematicallycomparing the direction of effect observed in the SuRE assay (raQTL activity change) with the predicted TF binding change. Concordant effects (activity change matches binding change) are most likely attributed to putative activators, while discordant effects (activity change opposes binding change) are most likely attributed to putative repressors. (**C**) Cell-type specificity of raQTLs based on TFBS enrichment. Enrichment analysis demonstrating that predicted TFBSs affected by raQTLs are specifically enriched for TFs relevant to the cardiac lineage (AC16 cells). r, the Pearson correlation coefficient.

One of the primary mechanisms by which a non-coding variant affects gene regulation is by creating or disrupting a TF binding site (TFBS)(24). Hence, we interrogated the genomic regions of raQTLs to predict changes in TF occupancy using JASPAR, a database of experimentally defined TF binding profiles stored as position frequency matrices (PFMs)(25). For a given raQTL and TF combination (an raQTL-TF pair), we calculated binding scores for the REF and ALT sequences. The corresponding p value for each score was computed as the probability that a random k-mer sequence has a better binding score than the sequence in question. By using the log of the p value ratio between the REF and ALT sequences, we calculated an effect size for each raQTL–TF pair. For instance, our analysis predicted a reduction in the binding of ELK3 to rs114081993 when the ALT allele was present compared to the REF allele (Fig. 2A). Intriguingly, the SuRE assay also revealed a similar decrease in activity at rs114081993 for the ALT allele (Fig. 2A). Additionally, the TFBS impact prediction analysis associated rs28804684 with REST, a known TF involved in gene silencing(26). More specifically, we predicted a decrease in REST binding and observed a corresponding increase in SuRE activity for the ALT allele at rs28804684 (Fig. 2A). Systematically comparing the direction of effect for all raQTLs (as indicated by SuRE) with the impact predicted by the TFBS analysis for TFs expressed in the AC16 cell line, we observed a high concordance (Fig. 2B), suggesting that indeed disruption of a known TFBS plays a major role in creating raQTLs.

The observation of cell type specific raQTLs(19) suggests that in part the TFs whose binding sites are affected by these raQTLs are likely to be expressed in a cell type-specific manner. To investigate this hypothesis, we focused on TFBSs that were significantly enriched among raQTLs in at least one cell type (AC16, K562, or HepG2) and subsequently compared the relative enrichment between these cell types. We found that the TFs whose motifs are specifically enriched in a cell line’s raQTLs exhibit significantly higher expression in that same cell line (Fig. 2C). For instance, we detected a notable enrichment of binding sites for key cardiac TFs, such as TBX20 and FOXC1, among AC16 raQTLs compared to those in HepG2 and K562 cells (Fig. 2C). This enrichment aligns with the high expression levels of TBX20 and FOXC1 in AC16 cells, emphasizing their importance in cardiac biology. FOXC1, for example, has been implicated in regulating early cardiomyogenesis and embryonic stem cell-derived CMs(27). Similarly, TBX20 plays fundamental roles in cardiovascular development, homeostasis, and cardiac remodelling in response to various stresses, contributing not only to heart development but also to adult heart function and adaptation(28). Conversely, binding sites for TFs crucial for hepatocyte function, such as HNF1A and HNF4A(29), are enriched among HepG2 raQTLs (Fig. 2C). Likewise, binding sites for the erythroid-specific TFs GFI1B and BACH2, which are highly expressed in K562 cells and play critical roles in haematopoiesis and erythropoiesis(30)^,^(31), are enriched among K562 raQTLs (Fig. 2C). Notably, when comparing AC16 and HepG2 raQTLs, BACH2 and GFI1B TFBSs exhibit no significant relative enrichment (Fig. 2C). Similarly, the binding site for HNF1A demonstrates no relative enrichment in the comparison between AC16 and K562 raQTLs. Unexpectedly, a slight enrichment in HNF4A TFBS is detected in K562 raQTLs when contrasted with AC16 raQTLs (Fig. 2C). This marginal enrichment might be attributed to the expression of HNF4A in K562 cells, albeit at a lower level(32). Taken together, these observations emphasize the context-specific nature of SuRE signals and their potential connection to cell-type-specific gene regulation.

### Prioritisation of candidate raQTLs highlights rs74330989 and defines a putative enhancer

We next aimed to incorporate information more directly related to the biological relevance of identified raQTLs. Specifically, using whole-genome sequencing data from parents of affected children, we investigated whether candidate regulatory variants followed a recessive inheritance pattern. Among the 18,201 raQTLs identified, 2,130 were heterozygous in unaffected parents but homozygous in affected children. These variants were subsequently evaluated against the gnomAD database(33) to assess their population frequency, applying a minor allele frequency (MAF) threshold of < 1%. Only a single raQTL met this criterion, however, further inspection revealed homozygous carriers in the reference population. Consequently, no raQTLs satisfied all criteria, suggesting that the genetic architecture underlying these cardiac phenotypes may be more complex, likely involving polygenic contributions and/or incomplete penetrance of individual variants.

**Figure 3:**
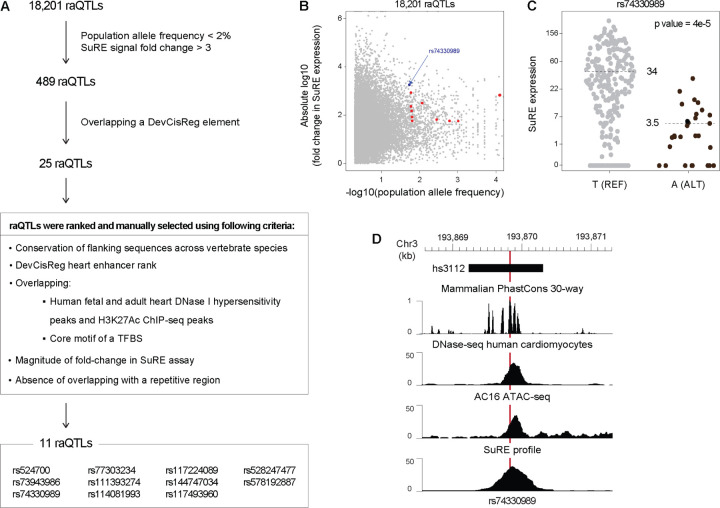
Prioritisation of candidate raQTLs highlights rs74330989 and defines a novel putative enhancer. (**A**) Candidate raQTL prioritization strategy. Schematic diagram outlining the multi-step strategy used torefine the list of 18,201 identified raQTLs down to the final candidates for in vivo validation. (**B**) Allelic effect ranking. Comparison of the 11 prioritized raQTLs (selected via the strategy shown in A;highlighted in red and blue) against all raQTLs identified. Note that rs74330989 exhibits the highest absolute fold change in SuRE regulatory activity between the REF and ALT alleles. (**C**) Allele-specific activity of rs74330989. SuRE activity levels for all genomic fragments encompassing rs74330989, grouped by the carried allele (REF vs. ALT). (**D**) Definition of the hs3112 enhancer. Integrative visualization used to predict and define the 1,073 bp putative hs3112 enhancer harbouring rs74330989. The definition is based on concordance across multiple data tracks, including: epigenomic marks (ATAC-seq and DHS), the SuRE activity profile, and sequence conservation across mammalian species. Epigenomic data were sourced from (35)^,^(100)^,^ (101).

Given this complexity, we next applied a broader strategy to prioritize variants with potential functional relevance using two filtering thresholds. First, we selected variants with a MAF of ≤ 2%, as reported in the gnomAD database(33); this more permissive cutoff (compared with the conventional ≤ 1% used above) was chosen to include variants with potentially incomplete penetrance. Second, to enrich for variants with stronger functional effects, we restricted the analysis to raQTLs showing a ≥ 3-fold difference in SuRE measurements between the REF and ALT alleles. Applying these criteria reduced the list of candidate raQTLs to 489 variants (Fig. 3A).

Next, we leveraged the comprehensive and continuously expanding Developmental Cis-Regulatory Elements (DevCisReg) resource, developed and maintained by Len Pennacchio and colleagues(34). This resource integrates diverse data types, including bulk H3K27Ac ChIP-seq, ATAC-seq, DNase I hypersensitivity (DHS)-seq, and, for certain organ systems, single-cell ATAC-seq (scATAC-seq) datasets. It defines enhancers spanning multiple organs and stages of embryonic development in both human and mouse. The underlying principle of DevCisReg is based on the observation that the magnitude of H3K27Ac enrichment of a peak within a sample correlates with the activity, biological importance, and in vivo validation rate of the corresponding enhancer. For each H3K27Ac dataset, peak magnitudes were calculated across open chromatin regions, ranked, and the top 15,000 peaks were retained. Notably, the DevCisReg resource includes extensive heart datasets, providing information on nearly 100,000 heart enhancers. An intersection of these established enhancers with 489 preselected raQTLs highlighted 25 candidate variants (Fig. 3A).

Then, the enhancer–variant pairs were manually inspected considering (i) the sequence conservation across 100 vertebrate genomes (PhastCons)(35), (ii) the DevCisReg heart enhancer rank, (iii) the relative magnitude of DHS in human cardiac tissues compared to other tissue types(36), (iv) the strength of H3K27Ac ChIP-seq peaks in both fetal and adult human left ventricles(37), (v) overlap core motif of a TFBS, (vi) the magnitude and statistical significance of allelic effects detected in the SuRE assay, and (vii) the absence of overlapping with repetitive elements or SNP clusters. This rigorous filtering process resulted in the selection of 11 enhancer–variant pairs that warrant further investigation. Here we focussed on rs74330989 as the most compelling candidate. This variant was found in a heterozygous state in the unaffected mother of the X38 family (Supplementary Fig. 1) and displayed the strongest allelic effect in the SuRE assay, showing a nearly 10-fold decrease in regulatory activity for the ALT allele compared to the REF allele (Fig. 3B,C). We defined the element of interest as the 1,073 bp region surrounding rs74330989 based on multiple lines of evidence including (i) ATAC-seq profile and SuRE activity measured in AC16 cells, (ii) DHS in CMs and (iii) high sequence conservation across vertebrates (Fig. 3D). The element, hereafter designated as hs3112, was selected for detailed functional characterization.

### Functional characterization and in vivo analysis of hs3112 and rs74330989

**Figure 4:**
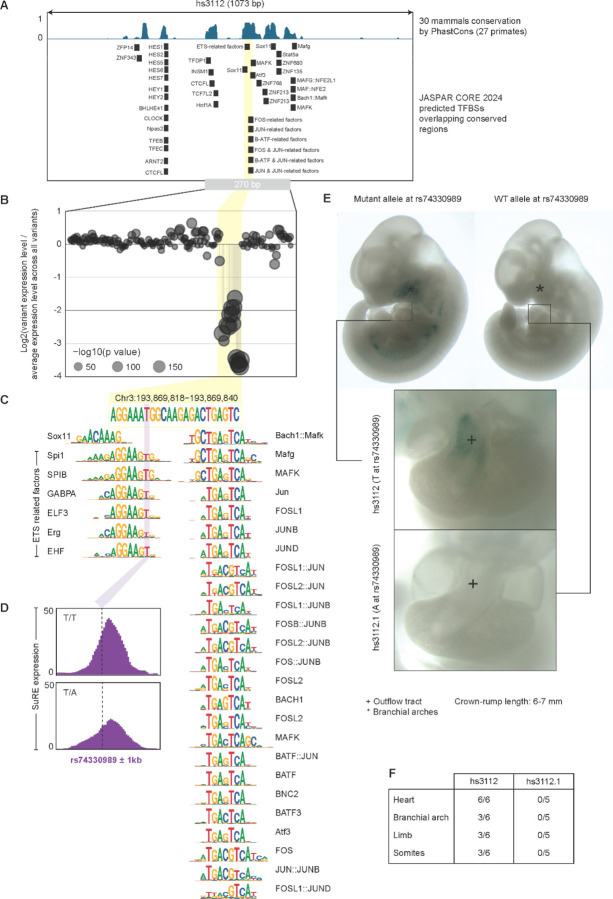
Functional characterization of the hs3112 enhancer and impact of raQTL rs74330989 on its activity. (**A**) Sequence conservation and TFBS prediction within hs3112. The hs3112 enhancer contains severalsmall, highly conserved domains (indicated by a PhastCons score across mammalian species near 1), within which binding sites for multiple TFs are predicted. The yellow-highlighted region marks the specific domain that overlaps the raQTL rs74330989. (**B**) Saturation mutagenesis defines the critical regulatory sites. Regulatory potential of all possible 1-bpdeletions within a 270 bp fragment of hs3112 containing rs74330989, as assessed by saturation mutagenesis combined with the SuRE assay in AC16 cells. A strong negative effect on regulatory activity is observed for mutations positioned immediately around rs74330989. (**C**) Impact of rs74330989 on key TFBSs. Two adjacent and critical TFBSs, one for ETS-related factorsand one for FOS/JUN/B-ATF heterodimers, appear essential for enhancer activity. The raQTL rs74330989 lies within the ETS-related factor binding site and disrupts the REF T allele. (**D**) Allele-specific reduction in activity. Comparison of SuRE signals from individuals homozygous(REF/REF) and heterozygous (REF/ALT) for raQTL rs74330989 reveals a significant reduction in reporter activity when the ALT allele is present. (**E**) In vivo validation of enhancer function and allelic effect. Validation of the hs3112 enhancer using theenSERT site-specific transgenic mouse assay. The wild-type enhancer drives robust LacZ expression in the developing mouse heart, brachial arches, limbs and somites at E11.5, whereas the rs74330989 mutation completely abolishes this activity. (**F**) Summary of LacZ reporter activity. Tabulated summary the presence or absence of LacZ reportersignal across the heart, branchial arches, limbs, and somites in the analysed mouse embryos for both wild-type and mutant hs3112 constructs.

We hypothesized that the hs3112 element functions as an heart enhancer and that rs74330989 influences its regulatory activity. Examination of the hs3112 sequence revealed several highly conserved hotspots across mammals, suggesting strong evolutionary pressure to preserve critical regulatory features (Fig. 4A). Closer inspection showed that these conserved segments are enriched in TFBSs (Fig. 4A; Supplementary Fig. 3), which is consistent with a predicted enhancer function(38)^,^(39). The rs74330989 is located precisely within one such conserved hotspot, which contains multiple TF binding motifs, including those for ETS-related factors, Sox11, and heterodimers of FOS, JUN, and BATF (Fig. 4A, highlighted in yellow).

To functionally annotate the importance of hs3112 subregions at high resolution in our model system, we performed a saturating mutagenesis experiment (Supplementary Fig. 3). The 1,073 bp hs3112 enhancer was divided into seven overlapping 270 bp fragments (with 135 bp overlaps), and single-nucleotide deletions were systematically introduced across each fragment. All mutant sequences, along with the wild-type controls, were used to construct a barcoded SuRE library, which was then transiently transfected into AC16 cells. This methodology allowed us to quantify the impact of every single-nucleotide deletion on regulatory activity using a pipeline analogous to the one described above for the whole genome libraries. Interestingly, not all conserved hotspots were intolerant to mutations, and some non-conserved regions displayed unexpectedly high functional sensitivity. Overall, we identified five regulatory subelements, ranging from 4 to 10 bp in length, mutations within which consistently affected the activity of the reporter gene. These included two repressive subelements (where deletions increased the activity, likely by disrupting repressor binding) and three activating subelements (where deletions decreased the activity, indicating essential bases for enhancer function) (Supplementary Fig. 3). The activating regulatory subelement containing rs74330989 and residing within the conserved segment showed the strongest effect of mutations (Fig. 4B). Within this 23 bp-long region, we observed two distinct clusters of impactful mutations corresponding to binding sites for ETS-related TFs and for FOS/JUN/BATF heterodimers (Fig. 4C). Specifically, rs74330989, which changes the REF T allele to the ALT A allele, is located within the ETS binding motif and leads to an ~ 50% reduction in the total regulatory activity of the locus in diploid heterozygous cells (Fig. 4D).

Importantly, evidence from mouse studies further supports the regulatory relevance of hs3112. Namely, ChIP-seq experiments have shown that key cardiac TFs, including Tbx5(40), Mef2c(41), Mef2a(42), Tead(43), and Srf(44), all critical for heart development(45), bind within the mouse counterpart of hs3112 in E12.5 mouse ventricle, underscoring its likely functional role in gene regulation.

To directly assess the predicted regulatory function of hs3112 in transgenic mice, we employed the enhancer inSERT (enSERT) system, a CRISPR/Cas9-based site-specific enhancer-LacZ reporter assay(34) (Supplementary Fig. 2C). We first tested the wild-type human hs3112 sequence in transgenic mice and indeed observed the LacZ reporter expression in the heart, branchial arches, limbs, and somites at embryonic day (E) 11.5 (Fig. 4E, top; Supplementary Fig. 4). Strikingly, introduction of the rs74330989-A mutation (construct hs3112.1) completely abolished enhancer activity; the LacZ signal was lost in all four tissues, with no detectable activity elsewhere (Fig. 4E, bottom; Supplementary Fig. 4). The observed activity patterns were highly reproducible across embryos collected for both wild-type and mutant constructs. Together, these findings demonstrate that hs3112 is a novel cardiac-specific enhancer, whose function strongly depends on the integrity of the TF binding motif hosting rs74330989.

### The hs3112 enhancer exhibits activity in NCC–derived lineages

**Figure 5:**
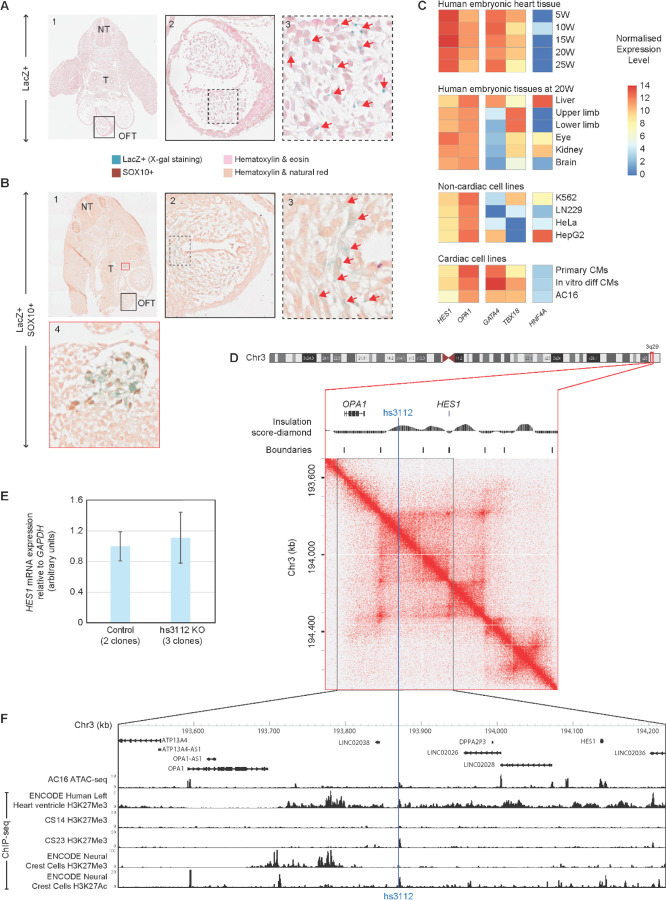
Potential regulatory role of the hs3112 enhancer in migrating NCCs during embryonic development. (**A**) Visualization of hs3112 enhancer activity. LacA staining in a heart-level transverse section from anE11.5 mouse embryo carrying a wild-type hs3112-driven LacZ transgene. Panels A2 and A3 are progressively higher-magnification views of A1, illustrating enhancer-driven expression in the OFT. (**B**) Co-localization of enhancer activity with NCC marker expression. Heart-level transverse section froman E11.5 mouse embryo carrying the hs3112-LacZ transgene and immunostained for Sox10. Panel B1 shows the full section; panels B2 and B4 sequentially higher-magnification views of B1, focusing on the OFT and the extracardiac sympathetic chain ganglia, respectively. Panel B3 is a further increased magnification of B2. (**A-B**) NT, neural tube; T, trachea; OFT, outflow tract. (**C**) Differential expression profiles of flanking genes. Human transcriptomic heatmap profile acrosscardiac and non-cardiac tissues, various developmental stages, and different cell lines. The heatmap displays expression levels of the two protein-coding genes flanking hs3112 (*HES1* and *OPA1*), along with known cardiac markers (*GATA4*, *TBX18*) and a contrasting liver-specific gene (*HNF4A*). *OPA1* shows broad, constitutive expression across all tissues and cell lines. In contrast, *HES1* is highly expressed in the embryonic heart but less so in other tissues, and its expression is lower in cell lines and differentiated CMs. Gene expression data were sourced from (80)^,^(88)^,^(89)^,^(90)^,^(91). (**D**) Chromatin architecture suggests *HES1* as the target. Hi-C heatmap from primary adult human left ventricle tissue suggesting that hs3112 and *HES1*, but not *OPA1*, reside within the same TAD, supporting a regulatory relationship. Hi-C data were sourced from (52). (**E**) Effect of endogenous hs3112 KO on *HES1* expression. *HES1* expression level measured by RT-qPCR shows no substantial change upon KO of endogenous hs3112 enhancer in AC16 cells. (**F**) Temporal and cell-specific repression of the hs3112 locus. While hs3112 resides in a region of openchromatin in cultured AC16 cells, this locus appears to be progressively repressed in most heart cell lineages during embryonic development, as indicated by the presence of the H3K27Me3 mark. Importantly, migrating NCCs and their descendants may represent an exception: the primarily active histone mark H3K27Ac is observed at hs3112 in in vitro differentiated counterparts of these cells. Epigenomic data were sourced from (101)^,^(53)^,^(55).

In vivo experiments using the LacZ reporter system revealed a distinctive inverted U-shaped domain of hs3112-driven activity within the embryonic mouse heart (Fig. 4E; Supplementary Fig. 4), closely resembling structures previously described in studies of migrating NCCs(46). After entering the pharynx, a subpopulation of NCCs migrates into the cardiac OFT cushions, where they contribute to septation and patterning of the great vessels and ultimately differentiate into smooth muscle cells of the arterial wall(7). In mice, at E9.5, NCCs arrive in the distal OFT and form two condensed mesenchymal “prongs” that later fuse via a dorsal shelf of mesenchyme to establish a horseshoe-shaped or inverted U-shaped septation complex(46) (Fig. 4E; Supplementary Fig. 4). As septation progresses (E11–E15 in mice; Carnegie stages (CS) 17–23 in humans), these prongs extend and spiral, forming the aorticopulmonary septum^19^/04/2026 18:03:00. The LacZ expression pattern observed in hs3112 transgenic embryos overlapped both temporally and spatially with these known NCC-derived lineages. This interpretation was corroborated by analysis of heart sections, which provided higher spatial resolution (Fig. 5A). To further confirm that hs3112 enhancer activity is restricted to NCC-derived lineages, we performed Sox10 immunostaining on sections from transgenic embryos. Sox10 is robustly expressed in migratory NCCs at various developmental stages(47); however, by E11.5, the stage at which LacZ expression was assessed, its expression in the OFT is largely downregulated as NCCs undergo differentiation(48)^,^(49). Therefore, our analysis focused on other NCC-derived cell types in which Sox10 expression persists at this stage, specifically the extracardiac sympathetic chain ganglia along of the dorsal aorta and Schwann cell precursors within the developing limb buds(48)^,^(50)^,^(51) (Supplementary Fig. 5). In both contexts, we observed near-complete co-localization of LacZ and Sox10, with the expected predominantly cytoplasmic and nuclear distributions of the respective signals. Taken together, these findings indicate that hs3112 activity is restricted to NCC-derived lineages, including within the OFT.

### Putative target gene of the hs3112 enhancer

The genomic context of hs3112 provides further mechanistic clues. The enhancer is located between two protein-coding genes, *HES1* and *OPA1* (Supplementary Fig. 6A), both expressed in fetal heart tissue. However, their expression patterns differ: *OPA1* is broadly and consistently expressed across embryonic tissues and cell lines, while *HES1* shows more specificity towards embryonic heart tissue (Fig. 5C; Supplementary Fig. 7A,C). Consistent with the predominantly heart-specific activity of hs3112, the heart-enriched expression of *HES1* suggests that it is the more likely regulatory target compared to *OPA1*.

The chromatin architecture provides additional support for *HES1* as the primary target of hs3112, although the most relevant available Hi-C dataset originates from primary adult human left ventricular cells(52). Notably, the Hi-C map show that hs3112 and *HES1* reside within the same topologically associating domain (TAD), with sub-TAD structures further strengthening their spatial proximity. By contrast, *OPA1* is spatially insulated from hs3112, reducing the likelihood of regulatory interaction (Fig. 5D; Supplementary Fig. 6B).

To investigate the in vitro regulation of *HES1* by hs3112, we generated an enhancer knockout (KO) in the AC16 CM cell line, the same model used for the original SuRE screening. Despite the near-tetraploid nature of these cells (Supplementary Fig. 9), all enhancer copies were successfully deleted across independent clones. Nevertheless, *HES1* mRNA levels remained unaltered (Fig. 5E; Supplementary Fig. 9), indicating that hs3112 does not contribute significantly to *HES1* expression in AC16 cells.

Examination of the local chromatin landscape provided a possible explanation for the lack of transcriptional response in the AC16 KO clones. Although *HES1* is expressed at low levels in adult-derived AC16 cells, the broader hs3112–*HES1* TAD identified in the adult left ventricle is enriched for H3K27Me3(53), a repressive histone modification(54) (Fig. 5F). Furthermore, ChIP-seq data reveal a progressive accumulation of the H3K27Me3 mark in the human fetal heart between CS14 and CS23(55) (Fig. 5D; Supplementary Fig. 10). These findings suggest that hs3112 activity may be temporally restricted to an early developmental window and is likely absent in differentiated CMs.

## Discussion

### Identification of non-coding variants potentially involved in cardiac function using the SuRE reporter assay

We employed the SuRE reporter assay in the AC16 CM cell line to interrogate 4.7 million variants across six individuals with a genetic predisposition to cardiac defects. This analysis identified 18,201 variants that significantly altered regulatory activity, raQTLs. Subsequent prioritization, including intersection with independent cardiac-relevant chromatin datasets, yielded 11 variants of particular regulatory interest. Among these, rs74330989 showed the largest fold change in SuRE expression upon introduction of the ALT allele and was therefore selected for futher functional characterization.

A key strength of the genome-wide SuRE strategy is the extensive dataset it produces, which provides a resource for assessing the functional consequences of numerous common variants. In addition, these data can be used to train computational models aimed at predicting regulatory effects of untested variants(56). Nevertheless, the size of such screening libraries necessitates the use of highly transfectable cell lines, which may not always represent optimal model systems. Consequently, future studies may benefit from restricting the analysis to a more focused set of variants (e.g., approximately 10,000 sequences), which can be synthesized in vitro as short (~ 300–600 bp) DNA fragments encompassing both REF and ALT alleles and subsequently subcloned to generate SuRE reporter libraries. These libraries could be then applied in in vivo reporter assays using delivery systems such as AAV or lentiviral vectors.

### hs3112 functions as an enhancer in NCC-derived lineages and is disrupted by rs74330989

We defined the 1,073 bp region surrounding rs74330989 as a regulatory element, naming it the hs3112 enhancer. Within this element, we identified a 23 bp functionally critical site encompassing the raQTL. Mutations within this site significantly reduced reporter transcript levels, confirming the variant’s regulatory role (Fig. 4B; Supplementary Fig. 3). This 23 bp sequence is highly conserved among mammals (Fig. 4A), indicating essential functional importance. Our analysis further suggests that rs74330989 may disrupt binding of key activating TFs, such as ETS-related factors, which are likely essential for hs3112 function across species (Fig. 4C).

To assess hs3112 function in vivo, we created transgenic mice carrying the human wild-type enhancer upstream of a LacZ reporter. At embryonic stage E11.5, the enhancer was active in the heart, branchial arches, limbs, and somites (Supplementary Fig. 4). Notably, it drove an inverted U-shaped LacZ expression pattern in the developing heart, closely mirroring the distribution of cNCCs, a population well characterized in mouse and chicken(57) cardiogenesis. Introduction of the single rs74330989-A variant into hs3112 abolished LacZ signal in all tissues (Supplementary Fig. 4).

Originating from the dorsal neural tube, NCCs migrate through the pharyngeal region and contribute to the aorticopulmonary septation complex, which divides the OFT into the aorta and pulmonary trunk(46)^,^ (58). Transverse sections of the transgenic embryos revealed hs3112 activity in a small number of cardiac cells, specifically within the outflow cushions, the expected anatomical location of cNCCs(59). Strong reporter activity was also observed in the branchial arches, the entry site for NCCs migrating toward the heart (Supplementary Fig. 5). Immunostaining for Sox10, a NCC marker, revealed co-localization with LacZ-positive cells in the extracardiac sympathetic chain ganglia flanking the dorsal aorta and in Schwann cell precursors within developing limb buds(48)^,^(50)^,^(51) (Fig. 5B; Supplementary Fig. 5). In contrast, Sox10 protein within the OFT was nearly undetectable (Fig. 5A), consistent with previously reported expression patterns at this developmental stage(47). Indeed, several studies have shown that Sox10 expression is progressively downregulated at both the mRNA and protein levels in cNCCs, particularly during differentiation toward smooth muscle lineages(48)^,^(49). Together, these observations indicate that Sox10 is not an optimal marker for identifying cNCCs in the OFT at E11.5. Nevertheless, robust co-localization of Sox10 and LacZ signals in other NCC-derived lineages supports the conclusion that hs3112-driven LacZ expression faithfully marks NCC-derived cell types. Accordingly, we infer that LacZ-positive cells within the OFT, despite lacking detectable Sox10, represent a subpopulation of cNCCs. Definitive identification of the specific NCC subpopulation marked by hs3112 will require lineage-tracing at earlier developmental stages, which is beyond the scope of this study.

Finally, although low levels of the repressive mark H3K27me3 were detected at the hs3112 locus in NCCs, this region exhibited a clear enrichment of the active chromatin mark H3K27Ac (Fig. 5F), suggesting that the enhancer is likely active in these cells and, consequently, in some of their descendant subpopulations. Notably, despite a generally repressive chromatin environment in adult tissues, ATAC-seq peaks remain detectable at the hs3112 locus in both AC16 cells and adult human left ventricle samples (Fig. 5F; Supplementary Fig. 10), indicating retained chromatin accessibility in at least a subset of the cells.

#### A potential role for hs3112 in HES1 regulation

The hs3112 enhancer is positioned between the *OPA1* and *HES1* genes (Fig. 5D), both of which are expressed during embryogenesis in multiple tissues, including the limb, brain, and liver. Notably, *HES1* exhibits substantially higher expression than *OPA1* in the embryonic heart. By contrast, *HES1* levels decline markedly at later developmental stages and in differentiated CMs, such as AC16 cells, whereas *OPA1* expression remains robust (Fig. 5C; Supplementary Fig. 7A). These observations indicate that *HES1* expression is largely confined to early developmental stages(60).

Several lines of evidence support a regulatory relationship between hs3112 and *HES1*. First, both loci reside within the same TAD in adult human left ventricular tissue, the most biologically relevant Hi-C dataset available (Fig. 5D), and a conserved chromatin architecture is observed in mouse CMs (Supplementary Fig. 6B). Second, chromatin profiling across fetal and adult cardiac samples shows that at early developmental stages (e.g., CS14), hs3112 is marked by both active/poised modifications (H3K4Me1/2/3) and the repressive mark H3K27Me3 (Fig. 5F; Supplementary Fig. 6D; Supplementary Fig. 10), consistent with a bivalent chromatin state or a mixed cell population. At later stages (e.g., CS23), the repressive mark predominates (Fig. 5F; Supplementary Fig. 6D; Supplementary Fig. 10), suggesting that enhancer activity is developmentally restricted. A comparable pattern is observed during mouse cardiogenesis (Supplementary Fig. 6C). It should be noted, however, that these datasets were generated from heterogeneous tissue samples; thus, signals from small cell subpopulations, such as cNCCs, may be masked. Third, there is spatiotemporal concordance between *HES1* expression in the fetal heart and NCC-specific activity of hs3112, including within cNCC derivatives. Consistently, previous studies have shown that *HES1* mutant mice display reduced NCC populations in the OFT(61). Fourth, the A17-Myf5-nlacZ-T55 (T55) transgene, integrated approximately 24 kb downstream of the hs3112 ortholog and 226 kb upstream of *Hes1* in the mouse genome (Supplementary Fig. 6B,C), is active during cardiogenesis in second heart field progenitors and their derivatives in the OFT myocardium. Its expression is also observed in the CNS, pharyngeal epithelia, pericardium, limb bud, and lung endoderm(62). The overall expression pattern has been attributed to a position effect at the integration site and largely overlaps with endogenous *Hes1* expression(61), suggesting shared regulatory control. Given the proximity of the T55 insertion to the hs3112 ortholog, this enhancer represents a plausible candidate and may serve as a primary driver of both T55 and *Hes1* activity.

Despite observing episomal enhancer activity of hs3112 in AC16 cells using the SuRE assay, as well as low but measurable *HES1* expression in this line, deletion of the endogenous enhancer did not result in a significant change in *HES1* levels (Fig. 5E). One explanation is that *HES1* is not the primary target of hs3112 in AC16 cells. Alternatively, the enhancer may be inactive in its native chromatin context, even though it appears functional in reporter assays when removed from potential positional repression. Collectively, these findings indirectly support the notion that hs3112 is epigenetically repressed in AC16 cells. Ideally, functional disruption should be tested in in vitro-differentiated NCCs, where hs3112 displays low H3K27Me3 and high H3K27Ac levels (Fig. 5F), consistent with active enhancer function.

### hs3112 as a candidate regulator of Notch–HES1 signaling in cNCCs

The hs3112 enhancer is enriched for predicted binding motifs of the HEY and HES TF families, including a site recognized by the transcriptional repressor HES1 (Supplementary Fig. 7B). In addition, HES1 is a well-established downstream effector of Notch signaling. Importantly, HES1 can negatively regulate its own transcription through promoter binding, forming an autoregulatory feedback loop that generates oscillatory expression patterns^63^. Such dynamics are central to the fine-tuning of Notch pathway activity and cell fate decisions(63). Collectively, these observations raise the possibility that hs3112 contributes to this regulatory circuitry during development, potentially facilitating *HES1* expression in migrating cNCCs before becoming repressed by HES1 itself.

The interaction between Notch signaling, *HES1*, and NCC biology has been extensively examined in the context of mouse cardiogenesis(64). Disruption of the pathway compromises cNCC migration and differentiation: for instance, Jag1-deficient mice lack NCCs in the OFT(65), while inactivation of *Notch2*, an upstream regulator of *HES1* and *HES5*, results in smooth muscle defects(66). Consistent with these findings, *HES1*-null mice display reduced NCC populations within the OFT, supporting a direct role for this factor in NCC development(61). Developmental expression analyses further show that *Hes1* is expressed in the OFT at embryonic day E8.5, preceding to NCC arrival and robust activation of canonical Notch signaling (Supplementary Fig. 7C), suggesting an early, Notch-independent phase. By E10.5, NCCs have populated the OFT and multiple Notch pathway components, including *Dlk1*, *Hes1*, *Hey1*/*2*, and *Notch1/2*, are expressed (Supplementary Fig. 6D). Given that hs3112 is active in migrating NCCs at E11.5, these observations point to a potential regulatory connection between the putatively bivalent hs3112 enhancer, *HES1*, and Notch signaling during cardiac development (Supplementary Fig. 11). Further studies will be required to directly test this model.

### Involvement of rs74330989 in disease regulation

The variant rs74330989 has a MAF of 1.8%, with 45 homozygous carriers reported in the refrence population database gnomAD(33) (v4.1.0), suggesting that it is unlikely to be a highly deleterious mutation capable of causing severe disease on its own. Consistent with this, the variant was identified in the mother of a child diagnosed with Cranio-cerebello-cardiac (3C) syndrome, a condition characterized by multiple congenital anomalies including CHD(67) and was absent in the affected child. The mother, who is heterozygous for the variant, displayed normal cardiac ultrasounds while the father did not have the variant. Together, these observations suggest that rs74330989 alone does not cause cardiac disease.

Despite this, our SuRE data indicate that rs74330989 has a strong functional impact on the hs3112 enhancer, and in vivo transgenic evidence suggests that this enhancer is active during early developmental stages in the heart. This raises the possibility that disruption of hs3112 could affect cardiac gene regulation. Extrapolating from mouse transgenic results, enhancer function might also be compromised in human carriers of the variant, yet the absence of a detectable phenotype in the mother implies that a single affected hs3112 allele may be not sufficient to significantly disrupt cardiac development.

It is also possible that other regulatory elements compensate for the loss of hs3112 activity. Indeed, enhancer redundancy is common across mammals and ensures robustness in gene regulation(68). Such compensatory mechanisms could maintain *HES1* expression during development despite disruption of a single regulatory element. Consistent with this idea, hs3112 may contribute only modestly to overall *HES1* regulation, so loss of its activity alone may not substantially perturb gene expression (Supplementary Fig. 11).

Importantly, our functional assessments focused on individual genetic variants in isolation. However, complex diseases often arise from the combined effects of multiple variants(69), and emerging evidence suggests that clinically “Mendelian-like” congenital disorder may involve contributions from multiple variants(70). In this context, regulatory variants may exert modest effects that become biologically significant only in combination. Future studies could explore approaches analogous to polygenic risk scoring within MPRAs, allowing the cumulative impact of multiple regulatory variants to be assessed.

Finally, although this study generated a large catalogue of regulatory variants and their functional effects in AC16 human CMs, future applications could adopt a more patient-focused approach using physiologically relevant cell models, including primary cells or induced pluripotent stem cell–derived cell types. For example, candidate variants identified in large cohorts of individuals with specific cardiac diseases could be prioritized, and the corresponding genomic fragments containing these variants could be synthesized in vitro and assayed using MPRA, avoiding the need for complex whole-genome libraries. Such strategies could provide a scalable means of evaluating the regulatory potential of patient-derived variants across diverse cellular contexts, thereby improving the interpretation of non-coding variants in clinical genomics.

## Conclusion

Using SuRE in human AC16 CMs, we functionally screened 4.7 million non-coding variants from six CHD-predisposed individuals and identified 18,201 raQTLs, generating a large resource for regulatory variant interpretation. Prioritization with rarity and developmental heart epigenomic datasets pinpointed rs74330989 and led to the discovery of a conserved 1,073 bp enhancer, hs3112, with a critical ~ 23 bp core likely mediated by TF binding. In transgenic mouse embryos (E11.5), hs3112 drives robust enhancer activity with a pattern consistent with neural crest–derived OFT lineages, and introducing the rs74330989 ALT allele completely abolishes enhancer activity in vivo. Genomic organization, chromatin context, and developmental expression patterns further support HES1 as a plausible downstream target of hs3112, implicating this enhancer in regulatory pathways linked to cardiac neural crest biology.

Together, these findings demonstrate the utility of large-scale functional assays for identifying developmentally relevant non-coding regulatory variants. More broadly, this work highlights the importance of integrating massively parallel reporter assays with developmental and epigenomic analyses to improve the functional interpretation of non-coding variation in human disease.

## Materials and Methods

### Patient selection from a larger CHD cohort

Due to technical and resource constraints, only six whole-genome SuRE libraries could be analyzed. Five patients were selected with hypoplastic left heart syndrome (HLHS), with parents showing normal cardiac ultrasound findings but a positive family history (at least one first-, second-, or third-degree relative with CHD), from for whom additional biological material from the proband was available for further analyses. A sixth patient did not have a heart defect, but was one of three siblings with an unclarified but identical syndrome characterized by dysmorphic features and intellectual disability; the other two siblings had septal heart defects.

### Preparation of SuRE libraries

SuRE libraries were generated following established protocols(19). Genomic DNA was isolated from blood samples obtained from six individuals provided by the University Medical Centre Groningen (UMCG). The isolated genomes were subjected to fragmentation and gel purification to yield fragments ranging from 400 to 600 bp (Supplementary Figure 1B). A separate SuRE library, with an approximate complexity of 600 million fragment–barcode pairs, was generated for each individual genome (Supplementary Table 1). Paired-end sequencing (2× 150 bp) of the resulting SuRE libraries was performed by Novogene using the Illumina NovaSeq 6000 platform.

### Cell culture and SuRE experimental setup

AC16 cells (Merck, SCC109) were cultured in DMEM/F12 medium (Capricorn Scientific, Ebsdorfergrund, Germany) supplemented with 12.5% FBS, 4 mM L-Glutamine, 15 mM HEPES, and antibiotics (100,000 Units/L penicillin, and 100 mg/L Streptomycin) at 37°C in a humidified incubator with 5% CO_2_. For each SuRE library replicate, a total of 100 million cells were transiently transfected using the Amaxa Nucleofector 2b (Lonza) with program A-033 (5 million cells per cuvette, loaded with 20 μg of plasmid DNA). Twenty-four hours post-transfection, cells were harvested for total RNA isolation. Subsequent sample preparation for NGS was performed as previously described(19). Single-read sequencing (50 bp) of the samples was performed by Novogene using the Illumina NovaSeq 6000 platform.

### Pre-processing of sequencing data

The paired-end reads generated from the SuRE libraries provided the association between genomic positions and barcodes for each fragment. Conversely, the single-end reads of the PCR-amplified barcodes quantify the expression level corresponding to each unique barcode. Both read sets were initially processed to remove adapter sequences and portions of the vector backbone using Cutadapt v4.3(71). Reads were subsequently discarded if the associated barcode sequence contained ‘N’ bases or if the sequence length was not precisely 20 bp. The single-end reads, which yielded the barcode sequences, were then counted and recorded to establish expression levels.

### Identification of variants

#### Variant calling strategy for SuRE libraries

Genome Analysis Toolkit (GATK) is a standard tool for genomic variant calling in both research and clinic settings. We adhered to the GATK best practice workflow for high-throughput sequencing data processing and variant discovery(72).

#### Aligning and mapping

The paired-end reads from each SuRE library were mapped to the reference human genome sequence (GRCh38.p13), including only chromosomes 1–22 and the X chromosome. Mapping was performed using Bowtie2 v2.3.4.1(73) with a maximum allowed insert size of 1 kb. Read pairs that likely originated from duplicates of the same original DNA fragment were marked as non-independent observations to prevent inflation of variant counts.

#### Variant calling and filtering

Germline short variants (SNPs, Insertions and Deletions) were discovered in individual patient genomes using GATK HaplotypeCaller(74). The following stringent thresholds were applied during variant calling and subsequent filtering. First, a minimum base quality score of 10 was required for a base to be considered for variant calling. Second, a minimum Phred-scaled confidence score of 30 was required for a variant to be officially called. GATK hard filtering was subsequently applied to all variants called by the HaplotypeCaller. Next, additional filtering based on allelic depths and allele frequency was performed, where a minimum read depth of 10 was required for each allele and an expected allele frequency between 0.4 to 0.6 was enforced at each variant site, consistent with heterozygosity. The filtered variant sites for each library were stored in a Variant Calling Format (VCF) file. Finally, the VCF files from all SuRE libraries were merged using BCFtools(75).

### Processing SuRE data

#### Linking variants, genomic DNA and expression

The initial analysis of the SuRE libraries, as described in the previous section, exclusively considered germline SNPs. To expand the analysis, we developed a novel pipeline that now incorporates the identification and assessment of small germline Indels (insertions and deletions). The pipeline is available at https://github.com/annogen/SuRE_Indel_pipeline.

#### Construction of pseudo-phased genome

The VCF file contains genotype information for each sampled position. The GT field within the VCF represents the genotype, which is encoded using allele values separated by either “/” (unphased) or “|” (phased). Allele values are defined as follows: 0 represents the REF allele; 1 represents the first ALT allele; 2 represents the second ALT allele, and so forth. Initially, all variant sites in the filtered VCF of each SuRE library were resolved to be biallelic. Specifically, only the two most frequent alleles across all patient samples were retained for subsequent analysis. A consensus sequence was generated using BCFtools consensus(76). For every variant positions the first allele (e.g., the allele corresponding to the first position in the GT field) was used to construct the first consensus sequence, and the second allele (e.g., the allele corresponding to the second position in the GT field) was subsequently used to construct the second consensus sequence (Supplementary Figure 12A,B). For a given library, these resulting consensuses sequences are referred to as the haplotype 1 genome and haplotype 2 genome, or collectively as the pseudo-phased genomes. Finally, the merged VCF file was lifted over to these newly constructed haplotype 1 and haplotype 2 assemblies to enable correct variant annotation in downstream analyses.

#### Aligning paired-end reads to pseudo-phased genomes

The pre-processed paired-end reads from each SuRE library were mapped to its corresponding pseudo-phased genomes (haplotype 1 and haplotype 2 assemblies) (Supplementary Figure 12C). Mapping was conducted using Bowtie2 v2.3.4.1(73). For each read pair, one of three classes was assigned based on its mapping and alignment quality (MAPQ) scores, as recorded in the Binary Alignment Map (BAM) file, relative to the haplotype 1 and haplotype 2 genome assemblies. Read pairs that were discordant (i.e., not a proper pair) were immediately discarded. All remaining reads for each library were assigned to one of the defined classes. The decision tree used for read assignment to the pseudo-genomes is illustrated in Supplementary Figure 12D. To enable correct annotation, the genomic positions of all variants were first lifted over from the original reference genome (GRCh38.p13) to both the haplotype 1 and haplotype 2 genome assemblies. Finally, reads in class 1 and class 2 were annotated by variants lifted over to the haplotype 1 and 2 genome assembly, respectively; reads in the “equal” class were annotated by variants lifted over to the haplotype 1 genome assembly.

#### Identification of raQTLs

The resulting associations between barcode sequences and genomic positions were first filtered to ensure data purity. Any barcode associated with reads belonging to more than one classification class (i.e., Class 1, Class 2, or “equal,” as defined previously) was removed from the analysis. Next, the expression level for each retained barcode was quantified by counting the number of reads corresponding to the reverse-transcribed complementary DNA (cDNA). This expression information was then integrated with the existing barcode sequence–genomic position pair and converted into a unified measure, referred to as the ‘SuRE count’. The identification of raQTLs was subsequently performed using a methodology analogous to that described in detail previously(19).

### TFBS impact (TFBSi) assessment

To assess the impact of raQTLs on TF binding, the JASPAR CORE (non-redundant) database was used, which provides a curated collection of 838 experimentally validated TF binding profiles from various eukaryotes(77). These profiles are represented as position probability matrices (PPMs) with motif widths ranging from 6 to 36 bp and an average length of 12.5 bp.

For each raQTL, a 31-bp region was defined, extending 15 bp upstream and 15 bp downstream of the variant. Two sequences were generated: the “reference element” and the “alternate element”, corresponding to the REF and ALT alleles, respectively. Motif detection was performed using the Find Individual Motif Occurrences (FIMO) tool from the MEME suite(78). Significant matches to all JASPAR profiles were identified using a stringent p value threshold of 1×10^−4^, and only motifs overlapping the raQTL position were retained for subsequent analyses.

To quantify TF binding affinity, each PPM was converted into a position weight matrix (PWM), which assigns log_2_-weight scores for motif matching. During this conversion, a small pseudocount (1×10^−10^) was added to avoid zero probabilities and ensure finite log values. Using the PWM, a binding score was calculated for each TF by summing the weights across all motif positions. This binding score was used as a measure of binding strength, and only TFs with a minimum score of 8 in either the REF or ALT sequence, or both, were retained to ensure high-affinity interactions.

The binding probability of each TF to the REF and ALT sequences was calculated using dynamic programming. By simulating binding across all possible k-mer sequences, the likelihood of TF binding to the sequence of interest relative to random sequences was estimated, generating separate p values for the REF and ALT alleles. TFs with a binding p value < 1×10^−5^ for at least one allele were retained. To quantify the effect of the raQTL on TF binding, the log_10_ ratio of REF to ALT binding p values was computed, reflecting the variant-induced change in binding affinity.

This analysis generated a list of TFs predicted to be disrupted by each raQTL, noting that a single raQTL can affect multiple TFBSs. TFs were then linked to their corresponding genes using the UniProt database(79). To ensure biological relevance, only TFs expressed in the cell type in which the raQTLs were identified were retained. For AC16 cells, genes with TPM > 1 in AC16 RNA-seq data(80) were considered expressed. This approach yielded a list of expressed TFs per raQTL predicted to be disrupted in the relevant cell line.

The relative enrichment of disrupted TFs between cell lines was assessed, based on the rationale that cell type–specific raQTLs may preferentially disrupt cell type–specific TFs. For each TF, the number of raQTLs affecting it in one cell line versus another was counted, and the log_2_ ratio of occurrences was computed. This value reflected the relative frequency with which a TF is disrupted in one cell line compared to the other. The relative expression of the genes encoding these TFs was then plotted in the same order. Relative gene expression was quantified as the log_2_ expression ratio between the two cell lines. Specifically, TFs affected by raQTLs identified in AC16 cells were compared with those from HepG2 cells, and separately with those from K562 cells.

Finally, the Pearson correlation was calculated between the log_2_ ratio of TF disruption frequency (i.e., the number of raQTL-affected binding sites in one cell line relative to the other) and the log_2_ ratio of expression of the corresponding TF gene between the same cell lines.

### Transcriptomic data analysis

RNA-seq quantification was performed using Kallisto(81). To prepare for mapping, an index file was first generated from the Gencode FASTA reference file(82). Quantification was conducted on the paired-end transcriptomic data from the LN229, K562, SH-SY5Y, HT1080, HepG2, and HeLa cell lines using 100 bootstraps and the default seed value of 42. Transcript-level abundances were subsequently aggregated at the gene level. Finally, expression values were normalized using DESeq2(83).

### Multi-tissue human embryo ChIP-seq data analysis

We conducted ChIP-seq analysis focusing on H3K27Ac ChIP-seq data from 13 distinct human embryonic tissues provided by the University of Manchester(84). The tissues analysed included brain, eye, palate, tongue, heart, lung, liver, pancreas, stomach, upper limb, lower limb, adrenal gland, and kidney. The ChIP-seq data processing adhered to the ENCODE “Histone ChIP-seq Data Standards and Processing Pipeline” guidelines(53). Reads were mapped to the hg38 reference genome using Bowtie2 with default parameters, resulting in BAM files.

Using the generated BAM files, peak calling was performed with Model-based Analysis of ChIP-Seq (MACS)(85) to identify H3K27Ac enrichment sites across the genome. Additionally, a BigWig profile was generated to facilitate the visualization of the data across the entire genome.

### A web-based tool SuREVizHeart

#### Implementation and application layout

SuREVizHeart was implemented in R v4.4(86) using the Shiny package. The interface is designed to be user-friendly, with a search box that accepts queries in the form of either a genomic variant (chr:pos format) or a gene name. To ensure robust data handling, the system displays an informative error message when unsupported input formats are provided, when variants have not been assayed by SuRE, or when genes are absent from GENCODEv75(82,87). Gene names are treated as case-insensitive to improve search flexibility.

Search parameters can be customized using a slider that defines the flanking region around the gene or variant of interest, ranging from 1 to 100 kb. Once a query is entered and validated, the corresponding data are displayed across multiple tabs. SuREVizHeart provides two main views: a variant view, used when a specific variant is queried (Supplementary Figure 13A), and a region view, used when a gene name is searched (Supplementary Figure 13B).

The application layout consists of a left-side menu bar with tabs corresponding to different data visualizations (Supplementary Figure 13C), and a search bar located at the top (Supplementary Figure 13D). Upon launching the application, a loading spinner is displayed in the main panel to indicate that the user input is being processed. The search bar includes three key buttons: Render, Download, and Browse (Supplementary Figure 13D). The Render button triggers data processing within the application; no analysis is initiated until this button is pressed. The Browse button allows users to upload MPRA, BigWig, and Browser Extensible Data (BED) files for visualization in the “Uploaded Track Viewer” tab. Multiple files can be uploaded simultaneously or in separate sessions, and uploaded files can be removed by clicking the remove icon next to each file name. The Download button enables users to download all SuRE data required to reproduce the visualizations shown in the application; however, additional published datasets used in the visualizations are not included.

#### Functional impact assessment

This tab enables evaluation of the functional consequences (as measured in the SuRE assay) of genetic variants within a specified genomic region. Two primary visualizations are presented in a single composite plot: the SuRE impact plot (Supplementary Figure 13A, top) and the gene plot (Supplementary Figure 13A, bottom). In the SuRE impact plot, variants are represented as rectangles, where the height reflects the difference in expression between the REF and ALT alleles. The width and opacity of the rectangles correspond to the statistical significance of this difference, with wider and darker rectangles indicating higher significance. This provides a visual representation of each variant’s functional impact on reporter gene expression. Below this, the gene plot displays all genes within the same region, with the queried variant highlighted for easy identification. An interactive feature allows users to click on any variant, recentering the plot on the selected position and enabling more detailed exploration of individual variants.

#### Variant data overview

This sub-tab (Supplementary Figure 13E) provides a detailed tabular summary of the variant data presented in the functional impact assessment tab, enabling closer examination of the variant’s effects on allele-specific expression in a structured format. Each column in the table is explained to facilitate interpretation. The queried variant is highlighted for easy identification. This view is intended to provide a more structured and in-depth presentation of the data for users who prefer tables over interactive plots.

#### Gene expression overview

This sub-tab (Supplementary Figure 13F) provides insights into the expression patterns of genes shown in the gene plot of the functional impact assessment tab. Expression is displayed across developmental stages, as well as across a range of tissues and cell types. Fetal heart transcriptomic data spanning post-gestational weeks 5 to 50(88) are included, along with tissue-specific expression data at week 25 (Carnegie stages 19–20)(89). In addition, a snapshot of fetal gene expression across 13 different tissues is provided. The tab also features gene expression data from mature CMs, derived from multiple sources, including primary adult human heart tissue(90), in vitro-differentiated CMs(91), and the AC16 CM cell line(80). Expression levels of TBX18 and GATA4 are highlighted as known cardiac developmental marker genes(92), while HNF4A is included as a liver-specific gene(93) to provide contrast.

#### Highlighted variants tab

This tab is divided into three sub-tabs, each providing different perspective on the functional impact of a selected variant by integrating functional, population-level, and clinical information.

##### Transcription Factor Binding Sites Impact (TFBSi):

This sub-tab (Supplementary Figure 13G) focuses on the effect of the variant on TFBSs. The impact is calculated based on changes in binding affinity between the wild-type and mutant sequences, using JASPAR(77) position weight matrix-derived binding scores. The resulting effect score reflects the extent to which TF binding, and consequently gene regulation, may be disrupted by the variant.

##### gnomAD:

This sub-tab (Supplementary Figure 13H) provides access to data from gnomAD(33), including allele frequencies, allele counts, and genotype quality across populations. It also incorporates in silico prediction scores, such as CADD(94) and SpliceAI(95), to help assess the potential functional consequences of the variant.

##### ClinVar:

This sub-tab (Supplementary Figure 13I) links to ClinVar(96) and provides information on clinical classifications, functional evidence, and relevant published studies. It is intended to clarify the clinical significance of the variant, including any known associations with specific diseases or phenotypes.

#### Custom data

This section contains two tabs designed to integrate external genomic data into the SuREVizHeart platform.

##### SuRE profile:

This tab (Supplementary Figure 13J) displays a static plot similar to that in the functional impact assessment tab, along with additional tracks for SuRE profile data, ATAC-seq data from the AC16 cell line, and PhastCons conservation scores across 30 mammalian species(97). These tracks provide broader context for interpreting the regulatory landscape surrounding a variant, highlighting conserved regions and areas of open chromatin.

##### Uploaded track viewer:

This tab allows users to upload custom MPRA data, BigWig, and BED files and visualize them alongside the SuRE data. This functionality enables users to compare their own datasets with SuRE results, facilitating customized exploration of genomic regions or variants of interest.

### Whole genome sequencing analysis of trio families

Adapter sequences were trimmed from 150bp paired end reads in all FASTQ files using Cutadapt v4.3(71). The trimmed reads were then aligned to the human reference genome (GRCh38.p13), including chromosomes 1–22 and X only. Alignment was performed using Bowtie2 v2.3.4.1(73) with a maximum insert size of 1 kb. Read pairs likely originating from duplicate fragments were marked as non-independent observations to avoid inflation of variant counts. Variants were initially called for each sample using HaplotypeCaller(74) in GVCF mode (–emit-ref-confidence GVCF). The resulting GVCF files were combined across samples using GATK GenomicsDBImport, followed by joint genotyping with GATK GenotypeGVCFs to generate a multisample VCF. SNPs, insertions, and deletions were thus jointly genotyped across all individuals. Variant filtration was performed using GATK VariantFiltration with threshold-based criteria. Variants were flagged if they met any of the following conditions: Quality by Depth (QD) < 2.0, Fisher Strand bias (FS) > 60.0, Mapping Quality (MQ) < 40.0, Strand Odds Ratio (SOR) > 4.0, Mapping Quality Rank Sum (MQRankSum) < −12.5, or Read Position Rank Sum (ReadPosRankSum) < −8.0. After filtering, only variants annotated with a PASS flag were retained using BCFtools, and these high-confidence variants were used for downstream analyses.

### Saturation mutagenesis of the hs3112 enhancer and data processing

Prior to mutagenesis, the 1,073 bp enhancer sequence (Supplementary Data 1) was divided into the following set of seven overlapping 270 bp fragments: 1–270 bp, 135–404 bp, 269–538 bp, 402–671 bp, 536–805 bp, 670–939 bp, and 804–1,073 bp. All possible single-nucleotide deletions were systematically introduced across each fragment in silico. The mutant sequences were synthesized by Twist and subsequently cloned into a barcoded SuRE plasmid backbone, following the methodology used for the whole genome SuRE libraries. The resulting plasmid library was transient transfected into AC16 cells as previously detailed, with the exception that a total of 10 million cells were used per replicate. The subsequent sample preparation and NGS were performed identically to the whole genome SuRE library protocol.

Data processing was performed using R v4.4(86). Raw reads were mapped to hs3112 locus in the hg38 reference genome assembly and quantified separately for each of the seven 270 bp tiles. Variant activity was quantified as the log_2_ ratio of each variant’s SuRE signal to the mean signal of all variants within the 270 bp tile. Statistical significance was assessed using −log_10_(p values) derived from a Wilcoxon test. Data were visualized per tile to map functional effects across the enhancer.

### Analysis of enhancer activities in vivo

Transgenic enhancer-reporter assays were performed essentially as described previously(23)^,^(98). Briefly, the wild-type and mutated sequences of the hs3112 enhancer (Supplementary Data 1) were synthesized as double stranded DNA by Twist Biosciences. These DNA fragments were cloned into the enSERT vector (Addgene, 139098) using Gibson assembly (New England Biolabs). Correct assembly of the constructs was confirmed using the Primordium long-read sequencing service. Each complete transgene reporter cassette, which included the enhancer sequence of interest, a minimal *Shh* promoter and a LacZ reporter gene, was flanked by sequences complimentary to the murine *H11* safe-harbour locus. This cassette was integrated into the mouse genome using CRISPR/Cas9-mediated homology-directed repair via pronuclear microinjection. Embryos were collected at E11.5 and genotyped to confirm the presence of the transgenic construct. Collected embryos were fixed with 4% paraformaldehyde (PFA) for 30 min with gentle rolling at room temperature. Following fixation, β-galactosidase activity was detected by staining with the X-gal reagent. Stained embryos were imaged on a Leica MZ16 microscope.

### Immunohistochemistry (IHC)

The procedures were performed mainly as previously described(99), with minor modifications detailed below. PFA-fixed moused embryos were processed and embedded in paraffin. Sections, 5 μm thick, were cut transversely through the heart, mounted on slides, and subjected to immunohistochemical staining. The primary antibody used was a rabbit polyclonal anti-SOX10 (1:100 or 1:200 dilution; Novus Biologicals, NBP2–57023). Detection was carried out using a two-step detection system with goat anti-mouse/rabbit horseradish peroxidase (HRP) (Brightvision, DPVB110HRP). HRP activity was visualized using the chromogenic substrate diaminobenzidine (DAB). Immunostained sections were subsequently counterstained with either hematoxylin or neutral red.

### CRISPR/Cas9-mediated hs3112 knockout in AC16 cells

Briefly, CRISPR/Cas9-mediated KO was performed, largely following previously published methods(19). The following single-guide RNA (sgRNA) sequences, targeting the regions immediately flanking the ends of the hs3112 enhancer, were utilized: 5′-GGAACACTTACGCCAGACTC-3′ and 5′-ACCACTGTTCATGCGGGACC-3′. Approximately 300,000 AC16 cells were transfected (as detailed above) with a total of 2 μg of plasmid DNA. The DNA mixture consisted of two distinct plasmids, each co-encoding the Cas9 protein and one of the individual sgRNAs, which were combined in equal proportions. A week post-transfection, individual clones were isolated and screened by PCR followed by agarose gel electrophoresis to identify successful KO events. The following primer pairs were used for screening: 5′-ATACCCACCCAGAGGGCACTGGAGG-3′ and 5′-TCCGGGGCTGTCTTATCCACGCTTG (flanking hs3112), and 5′-CCCTTCCCTTCGGCGGCTAATTTC-3′ and 5′-CAGGGACTATGGCAGGAAAAGAGTTGAAAC-3′ (internal to hs3112).

### Analysis of the effect of hs3112 knockout on *HES1* gene expression

RNA isolation and cDNA synthesis were performed as previously described(19) using random hexamers. Quantitative PCR (qPCR) was conducted on cDNA samples prepared from individual KO clones to evaluate the functional impact of the KO on *HES1* expression levels. The following primer pairs were used: 5′-TCCGGAGCTGGTGCTGATAACAGC-3′ and 5′-TAGCAGCCACCGGGGACGAGG-3′ specific for the *HES1* gene, and 5′-ACGTCGTGGAGTCCACTGG-3′ and 5′-CAGGGGTGCTAAGCAGTTGG-3′ specific for the *GAPDH* gene, which was used as a reference.

To analyze the allelic ratio at the rs77930285 SNP located within the *HES1* 5′UTR, a 229 bp fragment spanning this SNP was PCR-amplified from both gDNA and cDNA samples. The following primers were used (standard Illumina adaptors were included at the 5’-ends but not shown here): 5′-CCCCGTCTACCTCTCTCCTTGGTCCTG-3′ and 5′-ACGAGGAATTTTTCTCCATTATATCAGCTGGC-3. The resulting amplicons were subjected to paired-end sequencing (2× 150 bp) at Novogene using the Illumina NovaSeq 6000 platform. Sequencing reads from five AC16 clones (two controls and three hs3112 KOs) were aligned to the expected *HES1* 5′UTR sequence using the standard mapping procedure described above. Pileup files were generated to obtain base-level read counts. Allele-specific representation of rs77930285 in both gDNA and cDNA samples was quantified by calculating the proportion of reads mapping to the REF (T) and ALT (G) alleles.

## Supplementary Material

Supplementary Files

This is a list of supplementary files associated with this preprint. Click to download.

- supp260517.docx

## Data Availability

Raw SuRE sequencing data are available from the corresponding author upon reasonable request. SuRE count tables, BigWig files for visualization of SuRE data tracks in genome browsers, lists of raQTLs, and a table containing SuRE data for all 4.9 million SNPs are publicly available in the Open Science Framework repository, https://osf.io/pyh83/. The web-based platform SuREVizHeart for exploring and visualizing the functional impact of non-coding genetic variants analysed in this study is available at SuREVizHeart, http://195.114.233.102:3838/. Details of elements hs3112 and hs3112.1 are available in the VISTA Enhancer Browser, http://enhancer.lbl.gov. Information about DevCisReg is available at DevCisReg GitLab repository, https://gitlab.com/lotard/devcisreg2.
